# Prophylactic and Therapeutic Vaccination against Hepatitis C Virus (HCV): Developments and Future Perspectives

**DOI:** 10.3390/v1020144

**Published:** 2009-08-12

**Authors:** Marian E. Major

**Affiliations:** Division of Viral Products, Center for Biologics, Food and Drug Administration, Bldg29A/Rm1D10, 8800 Rockville Pike, Bethesda, MD 20892, USA; E-mail: marian.major@fda.hhs.gov; Tel.: +1-301-827-1881; Fax: +1-301-496-1810

**Keywords:** vaccine, immune responses, immunotherapy, T cells, neutralizing antibody

## Abstract

Studies in patients and chimpanzees that spontaneously clear Hepatitis C Virus (HCV) have demonstrated that natural immunity to the virus is induced during primary infections and that this immunity can be cross protective. These discoveries led to optimism regarding prophylactic HCV vaccines and a number of studies in the chimpanzee model have been performed, all of which resulted in modified infections after challenge but did not always prevent persistence of the virus. Therapeutic vaccine strategies have also been pursued in an effort to reduce the costs and side effects associated with anti-viral drug treatment. This review summarizes the studies performed thus far in both patients and chimpanzees for prophylactic and therapeutic vaccination, assesses the progress made and future perspectives.

## 1. Introduction

Hepatitis C virus (HCV) is an enveloped virus with a single stranded, plus sense RNA genome. The RNA (∼9.6 kb in length) consists of a ∼341-base 5′ non-translated region (NTR), a single open reading frame encoding all virus-specific proteins (∼3011 amino acids), and a 3′ NTR. Translation of the genome RNA is cap-independent and mediated by the 5′ NTR which functions as an internal ribosome entry site. The resulting polyprotein is cleaved, both co-and post-translationally by host and viral proteases. The structural proteins, which include the core and the envelope glycoproteins (E1, E2) are located at the N-terminus; the nonstructural (NS) components are located in the carboxy terminus two thirds and include p7, NS2-3 (a protease), NS3 (a serine protease and RNA helicase), NS4A, NS4B, NS5A and NS5B (an RNA-dependent RNA polymerase) [1,2].

Transmission of HCV is typically by the parenteral route, but per mucosal transmission such as sexual and maternal infant transmission do occur. Persistent infections caused by HCV occur in 70–80% of the acutely infected population, the majority of which will develop chronic hepatitis and will be at risk for cirrhosis, end-stage liver disease and/or hepatocellular carcinoma [3]. HCV is associated with 40–60% of chronic liver disease in the U.S. Of these patients, one third goes on to develop progressive fibrosis and cirrhosis [4] making hepatitis C the major disease leading to liver transplantation [3]. Hepatitis C is now the major cause of hepatocellular carcinoma (HCC) in Japan, having displaced hepatitis B and, according to the CDC, there are 8,000–10,000 deaths each year in the U.S. alone due to HCV-associated cirrhosis and cancer.

Significant progress has been made in the therapy of chronic hepatitis C. Current therapies with pegylated IFN-α and Ribavirin clear HCV infection in approximately 50% of cases, but treatment remains very expensive, requires 6–12 months of therapy, and carries a significant risk of serious side effects [5–7]. Currently, there are about 25,000 new HCV infections each year in the U.S. alone, making the development of a prophylactic vaccine against this virus imperative. An immunologic approach to treatment is also sought to either replace or enhance drug treatment. Major benefits in expenses and logistics would be gained if it were possible to treat a chronic HCV patient with 2–3 doses of a therapeutic vaccine as opposed to 6 to 12 months of combination IFN and drug therapy.

In this review the current understanding of immune responses that are induced during primary and secondary infections with HCV and the efficacy of protective immune responses induced with prophylactic vaccine candidates in pre-clinical studies using chimpanzees will be discussed. The feasibility and progress of immunotherapeutic studies in humans and non-human primates designed to treat patients chronically infected with HCV will also be discussed.

## 2. Genetic Diversity of HCV

In general, during replication of RNA viruses errors are randomly introduced into the genome by the RNA-dependent RNA polymerase (RdRp) and, due to the lack of proof-reading function in these enzymes, remain uncorrected. Many of these misincorporations are lethal for the virus and go undetected, others are silent, leading to a change in the nucleotide sequence but maintaining the amino acid residue. A third type of mutation changes the amino acid sequence. These non-lethal mutations have either no effect on virulence or confer an advantage to the virus such that it can replicate more efficiently, evade the host immune response or develop drug resistance. Thus, variant RNA genomes or polypeptide sequences can evolve establishing, in the case of HCV, the co-existence of several closely related but distinct viruses within a single host, referred to as a quasispecies population.

Nucleotide sequences of viral isolates have been studied extensively and six major genotypes (designated 1–6) have been defined, differing by 30–35% over the complete genome [8]. The greatest genetic variability is observed in the E1 and E2 glycoproteins and the NS5A region with higher sequence conservation observed in the NS3 and NS5B regions, the core gene and the 5′UTR [8]. Within each of the six major genotypes there exist more closely related subtypes (designated 1a, 1b, 2a, 3a etc.) differing from each other by 20–25% at the nucleotide level [8]. An unusually high degree of amino acid variation has been observed in the N terminus of the E2 protein [9,10]. This hypervariable region, referred to as HVR1, is located between aa 384–410 of the polyprotein (aa1–27 of E2). The HVR1 varies enormously both within any one isolate and between different genotypes [11,12]. Secondary structure predictions indicate the HVR1 of HCV is highly unstructured [13], which is consistent with a tolerance for the degree of sequence variation observed, and has been shown to be dispensable for replication and *in vivo* infection [14]. The HVR1 has also been shown to contain neutralizing antibody epitopes [15] and observations that specific antibody to this region changes during the course of chronic infections suggests that HVR1 is subject to immune pressure with the potential for escape mutants [13,16–18].

HCV sequences are continually evolving during an infection due to the error-prone NS5B RdRp, which generates an estimated 10^−5^ mutations/nucleotide/replication [19], and the high production and clearance rate of the virus, estimated at 10^12^ virions per day [20]. Immune escape has been shown directly and indirectly in a number of instances for natural infections in both T-cell [21–23] and B-cell [16,18,24] epitopes. Immune escape in CD4+ [25] and CD8+ [26] T-cell epitopes have also been shown in chimpanzees following failure of T-cell based vaccines, although immune escape from neutralizing antibody has not yet been demonstrated in vaccine studies.

HCV genetic diversity poses problems for vaccine development from the perspective of target antigens and the potential for escape from the vaccine-induced immune response. The variation in virus isolates has led to suggestions that a vaccine would need to be tailored to all six genotypes to be effective. However, patient plasma samples and monoclonal antibodies have been identified that are capable of cross-neutralizing a number of different genotypes [27–30] suggesting an effective cross reactive vaccine targeting the envelope region could be designed. T-cell-based vaccines have also received a great deal of focus partly due to the role of T-cells in natural clearance (see Section 4 below) but also because these vaccines can target the more conserved regions of HCV. Both of these approaches for the development of prophylactic vaccines against HCV are discussed more fully in Section 5.

## 3. HCV Kinetics During Primary and Secondary Infections

**Figure 1.**
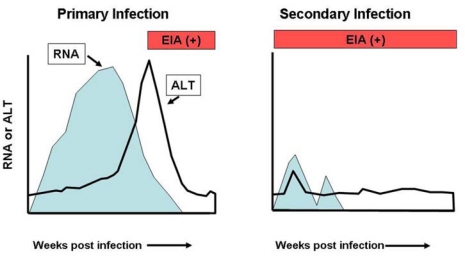
Schematic representation of primary and secondary infections with hepatitis C virus. Viral RNA titers in the serum are shown in blue; serum alanine aminotransferase (ALT) levels indicative of hepatitis are shown as a black solid line; seroconversion to anti-HCV antibodies as assessed by commercial assays is shown in red.

In primary HCV infections acute hepatitis develops at ∼8–14 weeks, when serum ALT levels increase and HCV-specific T-cells become detectable in the liver. The replication kinetics are characterized by a rapid (logarithmic) viral increase during the first 1–2 weeks, with a mean doubling time of ∼0.5 days, after which the viral increase slows to a mean half-life of 7.5 days [31]. Concomitant with ALT elevations viral titers rapidly decline at ∼8–14 weeks [31–33] at which point CD8^+^ T-cell responses and increased intrahepatic IFN-γ expression can be detected [31,32,34,35], which is associated with T-cell mediated immune responses. Early studies on HCV infections in chimpanzees and humans demonstrated that individuals that had recovered from a primary infection with the virus could be reinfected with homologous or heterologous strains [36,37]. However, subsequent studies in humans and chimpanzees have demonstrated that although reinfection does occur the viral kinetics are very different with immediate control of viral replication and rapid clearance in the majority of cases [38–40], although persistent infections following prior clearance can occur [41]. These data support the argument that adaptive immune responses are induced during a primary infection and that these responses can modify secondary infections and clear HCV more rapidly. Studies on viral kinetics have shown that the second infection is of short duration with a reduced viral titer in the blood and liver in comparison to the initial infection and reduced hepatic inflammation as indicated by alanine amino transferase levels (ALT) (Figure 1) [38,39]. Importantly, this level of protection also appears to be effective against other genotypes [42,43].

## 4. Immune Correlates of Viral Clearance

Both CD4+ and CD8+ T-cells have been shown to play a major role in clearance of HCV during primary and secondary infections with strong support for the involvement of immune responses to HCV NS3 in clearance of acute infection [44–49] (reviewed in [50]). After spontaneous recovery from HCV, resting HCV-specific memory T-cells can be detected in the peripheral blood of patients for decades, in many cases in the absence of detectable HCV antibodies [51]. HCV-specific cellular immune responses also persist in the liver, as demonstrated in recovered chimpanzees, and mediate protective immunity on reinfection [34,35,39,52,53]. The essential role of neutralizing antibody in controlling viral replication during primary or secondary infections is still unclear. Until the development of lentiviral/HCV pseudotype particle systems bearing the HCV envelope glycoproteins on the particle surface [54,55] the neutralizing ability of anti-envelope antibodies had been difficult to measure. More recently a cell culture system for HCV was developed using a genotype 2a virus [56,57] and although the 2a genotype virus remains the only genome able to robustly replicate and produce infectious virus *in vitro* this system has been used to generate chimeric viruses expressing the envelope proteins of all HCV genotypes [58–61]. Using these tools it has been shown that patients produce antibodies that neutralize infectivity *in vitro* and can cross neutralize a number of different genotypes [27,62]. High titers of neutralizing antibody are not produced until the chronic phase of the infection [27] and although the induction of neutralizing antibodies has in some cases been associated with clearance of the virus [63–65], it appears such antibodies may not be an absolute requirement for clearance [27,66]. Despite conflicting reports regarding the importance of antibodies to the surface glycoproteins of HCV during acute infections such antibodies have been shown to neutralize or control HCV RNA levels in a number of *in vivo* experiments [67–69] and neutralize virus or virus-like particles using *in vitro* systems [24,54,55,62,66] and should still be considered as an important component of any HCV vaccine.

## 5. Prophylactic Vaccines

A list of published HCV prophylactic vaccine studies in chimpanzees is shown in Table 1; these studies can roughly be divided into vaccines designed to induce T-cell responses, targeting non-structural proteins, or those primarily designed to induce neutralizing antibody responses, targeting the envelope region of the virus. The majority of vaccine studies have used homologous virus to challenge, i.e. the same genotype as that represented by the vaccine. In some publications the challenge virus is referred to as “heterologous” where a different variant of the same genotype has been used with approximately 7% diversity from the vaccine-based sequence. For consistency with terms of genetic diversity, in Table 1 “heterologous” has only been used to describe the challenge virus when a different genotype was used. This occurred in only one vaccine study performed by Folgori *et al*. [26] where the vaccine used genotype 1a sequences and the challenge was performed with a genotype 1b virus. Youn *et al*. [70] used a number of different genotypes to reinfect chimpanzees that had recovered from the primary challenge following vaccination. However, as these challenges followed clearance of a primary infection after vaccination, the extent to which the post-vaccine primary infection contributed to control or protection from the heterologous challenge can not be differentiated from the effect of the vaccine.

The majority of vaccine studies have used small numbers of chimpanzees (between one and six animals per study); however, important information can be gained from each one of these studies. Of particular importance is the fact that every prophylactic vaccine study published has successfully induced HCV-specific immune responses and has also led to modification of HCV replication soon after challenge, indicating that the vaccines have been effective at priming antibody or T-cell responses that can inhibit viral replication. However, despite this initial control persistent infections have developed in a number of the vaccinated chimpanzees. The persistence in some cases has been associated with immune escape in CD4+ [25] or CD8+ [26] T-cells and with higher viral mutation rates [71], suggesting that in cases where the immune response cannot rapidly clear the virus there remains an environment for selective pressure. Interestingly, the vaccines that seem to have had the greatest success at protection or leading to resolved infections have included all or part of the HCV envelope region inducing primarily either neutralizing antibody [72–74] or E1E2 T-cell responses [70,75]. These data suggest that neutralizing antibodies can play a role in protection but also that this region may contain T-cell epitopes that are important for clearance.

A major question surrounding HCV prophylactic vaccines is what should be the expected outcome following exposure in vaccinated individuals? Is sterilizing immunity a realistic goal or should rapid control of viral replication followed by clearance be an acceptable endpoint? Given our current knowledge of viral kinetics in secondary infections and results of a relatively large number of different vaccine studies in chimpanzees it appears that sterilizing immunity may not be achievable or guaranteed in 100% of cases. It has been shown that a number of well-established, highly important, licensed vaccines do not confer sterilizing immunity (e.g. measles [80], influenza [81], polio [82]) in that viral replication can be shown in vaccinees following exposure using cell culture or antigen studies. However, it has been clearly demonstrated that these vaccines induce immune responses that protect against illness, reduce the level of viral replication and have been enormously successful at controlling disease. The current methods that have been developed for detecting HCV in plasma are PCR-based assays that can detect very low amounts of virus, such as the transcription-mediated nucleic acid amplification assay (TMA) which can detect as few as 50 HCV RNA copies/mL [83]. These are far more sensitive than assays that were in place many years ago when most of the current vaccines for viral infections were developed. For example, when the hepatitis A (HAV) and hepatitis B (HBV) vaccines were licensed PCR testing had either not been developed or was in its infancy. In the pivotal clinical trials performed for the two main HAV vaccines currently on the market (Havrix® and VAQTA®) a case was defined by the presence of typical signs and symptoms, a diagnostic increase in IgM antibody to hepatitis A, and elevated serum concentrations of alanine aminotransferase [84,85]. HBV vaccines have been commercially available since 1982, well before PCR techniques were developed, with vaccine failures defined as the presence of surface antigen or core antibody or the absence of surface antibody [86]. Only recently in clinical trials for Human Papilloma virus (HPV) vaccines was PCR technology used to detect viral DNA but persistent HPV-16 infection was defined as the detection of HPV-16 DNA in samples obtained at two or more visits, with test samples obtained at enrollment, one month after the third vaccination, and every six months thereafter [87]. Therefore, in this most modern of vaccines designed to protect against a persistent viral infection transient viral replication was either acceptable or would have been missed. Similarly, preventing chronic infection with respect to HCV in the absence of sterilizing immunity may be an acceptable goal for a vaccine as in humans it is chronic persistence of the virus that is mainly associated with pathogenesis and the development of serious liver conditions [88,89].

### Antibody Based Prophylactic Vaccines:

The most comprehensive prophylactic vaccine studies have been performed by the Chiron/Novartis group led by Michael Houghton. Using an oil/water adjuvanted HCV envelope gpE1/gpE2 vaccine designed to induce neutralizing antibodies they have been able to compare the outcome of infection in a relatively large number of vaccinated chimpanzees (n=21) versus untreated controls (n=24) [72,73]. This recombinant vaccine was shown to elicit specific antibody and T cell responses. In animals with high anti-envelope antibody titers apparently sterilizing immunity was achieved, as assessed by RT-PCR, but even in vaccinated animals where virus could be detected by RT-PCR in the plasma post challenge the outcome of infection in vaccinees was more likely to result in clearance than persistence compared with untreated controls (p=0.002). Similarly, chimpanzee studies by Forns *et al*. [74] using a DNA plasmid to induce anti-E1E2 immune responses found modified infection and rapid clearance of HCV associated with high anti-envelope antibody titers. Furthermore, Puig *et al*. [76] showed that coupling a vaccine-induced anti-E1E2 antibody response, using recombinant gpE1/gpE2, with natural immunity resulting from clearance of the primary infection in a chimpanzee led to a subclinical infection upon rechallenge [76]. These data provide the most promising indications that antibodies to gpE1/gpE2, possibly coupled with T cell responses, can be protective by preventing or modifying HCV infection such that the virus can be cleared. However, although a number of neutralizing epitopes in E1 and E2 have been identified [28–30,55] and neutralizing antibodies have been raised in vaccinated chimpanzees [76,78,90] whether sterilizing immunity is consistently possible or what levels of *in vivo* titers are required to achieve either sterilizing immunity or successful modification of infection such that clearance is always achieved is still unknown.

The greatest challenge to neutralizing antibody-based vaccines when using a monotypic antigen for immunization is the generation of heterologous protection that can neutralize more than one genotype of HCV. Zhang *et al*. [90] recently showed that heterologous neutralizing ability is present in plasma from chimpanzees vaccinated with monotypic antigen but cross-neutralization could only be revealed when interfering antibodies specific to an epitope adjacent to a conserved neutralizing epitope in E2 were blocked such that the antibodies specific to the conserved region could be functional [90,91]. These findings have significant implications for development of an E1E2-based vaccine suggesting that the removal of specific immunogenic but non-neutralizing regions from a recombinant E1E2 vaccine could lead to the induction of a cross-neutralizing immune response. Further studies of this type need to be performed analyzing antibody profiles induced following vaccination and natural infection in order to dissect out which antibodies are effective for neutralization and which are immunogenic but non-neutralizing.

### T-cell based Prophylactic Vaccines:

Since the demonstration that T-cells are important for clearance of HCV a great deal of focus has been placed on developing vaccines that will induce T-cell responses to HCV proteins. These types of vaccines have an advantage over vaccines that focus on inducing immune responses to the envelope proteins as more conserved regions of the virus can be targeted potentially generating a more heterologous immune response capable of protecting against a number of HCV genotypes.

A number of approaches have been used to generate T-cell responses against HCV antigens in chimpanzee studies (Table 1); these include the use of virus-like particles and defective or attenuated viral vectors with or without priming of the immune system with DNA plasmids. The use of HCV virus-like particles produced in insect cells has proved successful at inducing effective HCV-specific immune responses, although interestingly the responses to the structural proteins were mainly T-cell specific with little or no neutralizing antibody detected [75]. All of the chimpanzee vaccine studies published to date have been successful at inducing specific immune responses to the target antigens and all have resulted in modification of the HCV infection and early control of replication following challenge with virus. The HCV-specific T-cells induced have been shown to produce IFN-γ and in many cases additional cytokines such as IL-2 and IL-4. However, eliciting broad CD4+ and/or CD8+ T-cell responses has not always prevented persistent infections in the animals studied. In some cases the development of persistent infections have been associated with lower immune responses compared to other vaccinees [26,77] or higher levels of viral mutation rates and immune escape [25,26,71]. Although it is difficult to quantitatively compare T-cell assays between different groups and laboratories the vaccine studies performed using the chimpanzee model seem to have induced more robust immune responses than those routinely detected in recovered patients or chimpanzees suggesting the quality of the immune response is more important than a quantitatively strong immune response for rapid clearance of the virus. These findings emphasize the importance of the chimpanzee model for testing the efficacy of a vaccine-induced response before proceeding to clinical trials. In the next generation of vaccine studies detailed analyses need to be performed of T-cell phenotypes induced and functional markers of protection such as homing profiles, central and effector memory phenotypes, T helper function and proliferation using markers such as CCR7, CD28, CD62L and CD45RA (reviewed [92]). It is possible that in cases where T-cell vaccines fail to clear the virus the induced HCV-specific cells are only secreting a single cytokine, such as IFN-γ, while it is necessary to induce polyfunctional cells that secrete multiple cytokines, such as IL-2 and TNF-α, as has been achieved with HIV vaccines [93]. These types of studies require large numbers of cells but are necessary before vaccines move into clinical trials involving any populations at risk for infection with HCV.

The types of viral vectors used for HCV T-cell vaccines are common to a number of strategies used for other vaccine targets such as HIV and include adenovirus [26,71,78] and vaccinia virus [25,70,77,79] vectors. Transferring these viral vectors to human clinical trials may be challenging due to pre-existing immunity or adverse side effects. Adenovirus vector modifications have been extensively explored in the vaccine field and include the use of alternative serotypes and genetic modifications [26,94]. In addition, a highly attenuated strain of vaccinia virus (Modified Vaccinia Ankara, MVA) has been developed which has proved to be immunogenic and as effective at eliciting immune responses as wild type virus [95,96]. The studies in chimpanzees to date have mainly been performed in order to establish whether a vaccine-induced T-cell response can be effective at modifying HCV infection and replicating the type of viral kinetics seen in recovered/rechallenged animals. A greater number of vector and delivery systems have been explored in animal systems other than the chimpanzee model and have been successful at inducing HCV-specific immune responses. Replication-defective alphavirus particles have been used in mice to induce NS3-specific cytotoxic T-cell responses [97] or in combination with Th1-adjuvanted proteins to induce neutralizing antibodies as well as broad CD4+ and CD8+ T-cell responses to non-structural proteins [98]. Alphaviruses are attractive as gene delivery systems due to high levels of foreign gene expression [99]; dsRNA intermediates, which can stimulate the innate immune system [100]; and the potential for cross presentation of antigen due to cytopathic effect. However, there are concerns regarding safety of Venezuelan equine encephalitis virus replicons and the replication levels and immunogenicity of Sindbis virus in humans [101]. An adenovirus vector-based minigene vaccine encompassing four domains of the NS3, NS4 and NS5B proteins that contain multiple class I/II restricted epitopes also induced strong and broad HCV-specific T-cell responses in HLA-A2 transgenic mice [102] and may prove promising as a tool for inducing cross-reactive responses. However, as challenge experiments have not been performed with these newer systems to determine efficacy caution needs to be taken in assessing potential success following scale-up to chimpanzees or humans in the clinical setting.

## 6. Therapeutic Vaccines

Great progress has been made in the therapy for HCV but the current standard of care for chronic infections still remains a combination of pegylated IFN-α and Ribavirin for 6–12 months. Previously clinical results showed an almost 100% sustained viral response rate in patients when treatment is initiated early (89 days) after infection [103] as compared with 50% of cases in the chronic phase [7]. However despite promising results for the treatment of new infections there still remains the problem of treating the ∼170 million chronically infected patients worldwide, many of whom have not responded to IFN/Ribavirin treatment in the past. With the advances in cell culture systems to study HCV replication [56,57,104,105] there has been a great deal of focus on the development of drugs to specifically inhibit viral enzymes such as the protease; helicase; and RNA-dependent RNA polymerase. In addition there is a role for therapeutic strategies to enhance HCV-specific immune responses that may be used alone or in conjunction with existing or new antiviral therapies.

A number of diverse therapeutic vaccine trails have been performed; these are listed in Table 2. Unlike prophylactic vaccines these have been carried out in HCV infected patients or healthy volunteers in a clinical setting. The approach of therapeutic vaccines has been similar to prophylactic vaccines in that the major goal has been to enhance HCV-specific T-cell responses although enhancement of envelope antibody responses has also been included in a number of studies. The strategies have included using recombinant proteins (core or E1) adjuvanted with ISCOMATRIX or alum. ISCOMATRIX is an adjuvant that forms a cage-like structure resulting in the induction of antibody and T-cell responses. Other strategies have used peptide vaccines; DNA vaccines, MVA vectors, and heat-inactivated antigens from HCV-infected donors. The current therapeutic vaccine trails to date have not succeeded in clearing HCV infections or achieving sustained reductions in viral titers. However, the studies have all successfully stimulated HCV-specific immune responses with transient reductions in viral RNA titers [106–108] and possible stabilization or improvement in liver histology or liver enzymes [109–111] in subsets of patients. Recently, the therapeutic peptide vaccine IC41 was added to pegylated IFN/Ribavirin treatment [112] in an attempt to decrease the relapse rate in patients receiving standard therapy. Although the IC41 vaccine did not prevent HCV-RNA relapse, specific T-cell responses were induced and were associated with lower relapse rates.

In addition to human therapeutic vaccine trials some *in vitro* studies have been performed using peripheral blood mononuclear cells from HCV infected patients [113,114]. One study used synthetic self-adjuvanting lipopeptides containing a CD4+ helper epitope and an NS5B epitope from HCV together with a ligand for Toll-like receptor 2 on dendritic cells (DCs) [113] for DC-based immunotherapy of HCV infection. The lipopeptides were able to activate monocyte-derived DCs from both healthy and HCV infected patients, the pulsed DCs were also able to activate autologous CD8+ T-cells from an HCV patient. A second study used modified adenovirus recombinants expressing core and NS3 of HCV [114]. The recombinant was able to activate and expand HCV-specific T-cells from HCV-infected donors *in vitro*. These strategies have yet to be tested in patients for effects on HCV viral titers but both have promising implications for immunotherapy.

Some strategies used for therapeutic vaccines (*e.g.* peptide or DNA vaccination) may not be as effective in naïve patients for inducing a *de novo* immune response compared to persistently infected patients where the immune system has already been primed by the natural infection. However, the same or similar viral vectors, such as MVA, could in principle be used for both types of vaccination strategies. Approaches such as the use of inactivated HCV antigens derived from HCV-infected donors may be considered too high a risk for use in healthy individuals as a prophylactic vaccine although if this proved effective at reducing HCV titers or clearing the virus recombinant forms of the proteins could be considered for future development. As more information is gained about possible immune dysfunction that may occur in persistently infected patients, therapeutic vaccines may need to be tailored to include specialized immunostimulatory molecules such as ligands for Toll-like receptors or vectors that secrete cytokines shown to be poorly expressed by T-cells from chronically infected patients.

The enhancement of HCV-specific immune responses in chronically infected patients is encouraging but further investigation and follow-up of existing clinical trials is required to determine if changes in the observed parameters in treated patients are sustained; if there is immune escape of the circulating virus from the boosted immune response and whether this may contribute to the transient reductions in virus titers; and what impact these strategies may have on patient outcome if combined with current or newly developed antiviral therapy.

## 7. Conclusions

In conclusion, the prospects for HCV vaccines are better today than they have ever been. This is partly due to overall advances in vaccine technology and methods to analyze and characterize immune responses but it is also due to advances in our specific understanding of natural HCV immunity, HCV replication and kinetics and the development of new tools to test antibody immune responses such as pseudotype particles and cell culture grown virus. However, there is still a great deal of missing information. We still do not know which types of immune responses correlate with protection or clearance of HCV during natural infection or the types of immune responses that are present during chronic infections that seem to exert only partial control of HCV. New vaccine studies also need to include higher level immunological analyses examining multifunctional activities of T-cells and T-cell phenotypes. It is still too early to assess how cross protective any vaccine candidate may be, mainly due to the difficulties of challenging large numbers of chimpanzees with multiple heterologous genotypes. Our current understanding from chimpanzee rechallenge studies and human exposure studies certainly indicates that natural cross protective immunity is present following a single genotype infection, suggesting that a multivalent vaccine tailored to all six genotypes would not be necessary. The development of a small animal model that is immunocompetent would advance the field enormously although care should always be taken in extrapolating data to humans as many immune response studies in mice have not translated well to humans and primates. We also need to investigate the effects of vaccine-induced immune responses on immune escape and possible persistence of HCV; this applies to both prophylactic and therapeutic vaccines.

## Figures and Tables

**Table 1.** Prophylactic HCV Vaccine Studies in Chimpanzees.

**Vaccine** **(Genotype sequence used)** **(Number of animals vaccinated)**	**Immunogenicity**	**Challenge** Inoculum* Dose (CID50§)	**Outcome**	**Ref.**
Recombinant gpE1/gpE2 in Oil/water adjuvants (Genotype 1a) (N=21)	Induced antibodies to E1E2	Genotype 1a Homologous 10 to 100CID50	Protects against infection or chronic infection 5 protected from infection 14 resolved infection 2 developed persistent infection	[72] [73]
DNA vaccine expressing E2 protein (Genotype 1a) (N=2)	Induced antibody and T cell responses to E2.	Genotype 1a Homologous 100CID50	Modifies infection, protects from chronic infection 2 resolved infection	[74]
Recombinant gpE1/gpE2 in oil/water adjuvants (Genotype 1a) (N=1)	Induced antibodies and cellular responses to E1E2	Genotype 1a Homologous 100CID50	Delayed/modified infection 1 developed persistent infection	[76]
DNA prime and protein boost (using C, gpE1, gpE2 and NS3) (Genotypes 1a (core, NS3) and 1b (core, E1E2, NS3)) (N=2)	Induced specific T-cell responses and antibody to E1 and E2.	Genotype 1b Homologous 25CID50	Modifies infection, protects from chronic infection 1 resolved infection 1 developed persistent infection	[77]
DNA prime and Recombinant Adenovirus expressing core, E1,E2 and NS3 to NS5B (Genotype 1b) (N=6)	Induced specific T-cell responses and anti-E2 antibody (neutralizing)	Genotype 1b Homologous 100CID50	Modifies infection, protects from chronic infection 1 protected from infection 1 resolved infection 4 developed persistent infection	[78]
DNA prime and Recombinant VV expressing NS3,NS5A,NS5B (Genotype 1a) (N=1)	Induced specific T-cell responses	Genotype 1a Homologous 100CID50	Modifies infection 1 developed persistent infection	[25]
DNA prime and Recombinant Adenovirus expressing NS3 to NS5B (Genotype 1b) (N=5)	Induced specific T-cell responses	Genotype 1a Heterologous 100CID50	Modifies infection, protects from chronic infection 4 resolved infection 1 developed persistent infection	[26]
DNA prime and rMVA boost (using C, gpE1, gpE2 and NS3) (Genotype 1b) (N=4)	Induced specific T-cell responses and antibody to E1 and E2.	Genotype 1b Homologous 25CID50	Modifies infection, protects from chronic infection 1 resolved infection 3 developed persistent infection	[79]
Recombinant VLPs containing C, E1 and E2 (Genotype 1b) (N=4)	Induced specific T-cell responses. No detectable anti-E1E2 response.	Genotype 1b Homologous 100CID50	Modifies infection, protects from chronic infection 4 resolved infection	[75]
Recombinant VV core, E1, E2, p7, NS2 and NS3 (Genotype 1b) (N=4)	Induced specific T-cell responses. Weak anti-E1E2 response.	Genotype 1b Homologous 2.5 and 24CID50†	Modifies infection, protects from chronic infection 4 resolved infection	[70]
DNA prime and Recombinant Adenovirus expressing NS3,NS5A,NS5B (Genotype 1a) (N=2)	Induced specific T-cell responses	Genotype 1a Homologous 100CID50	Modifies infection or protects from chronic infection 1 resolved infection 1 developed persistent infection	[71]

* Homologous and heterologous refer to the same or different genotype, respectively;

§ CID50=50% chimpanzee infectious doses;

† Animals erroneously received 2.5CID50 which did not lead to infection in control animals. Animals were then challenged with 24CID50.

**Table 2.** HCV Immunotherapeutic Vaccine Studies.

**Vaccine** **(Genotype sequence used)**	**Patient HCV** **Genotype**	**Immunogenicity**	**Outcome**	**Ref.**
Alum-adjuvanted recombinant gpE1 protein (Genotype 1b)	*Genotype 1a Genotype 1b	Antibody and T-cell responses in some healthy volunteers. Boosts humoral and cellular responses in a subset of patients	May ameliorate hepatitis	[109] [115] [116]
Peptide vaccine targeting E1, E2, NS3 and NS5A. (Genotype 1b)	Genotype 1b	Boosted peptide-specific T-cell and antibody responses.	Decreased ALT levels and HCV RNA levels in a subset of patients.	[106]
DNA vaccine (CIGB-230) expressing core, E1, E2 plus recombinant core protein (Genotype 1b)	Genotype 1b	Boosted neutralizing antibody responses and T-cell responses in patients.	May ameliorate hepatitis	[110]
Synthetic peptide vaccine (core, NS3, NS4) IC41 with or without poly-L-arginine. Genotype 1	Genotype 1 Genotype 3 Genotype 4	Boosted T-cell responses in patients.	Transient decline (>1- log) in HCV serum RNA titers in a subset of patients.	[107]
V-5 Immunitor – heat-inactivated HCV antigens from HCV-infected donors administered orally in tablet form. (Genotype not defined)	Genotype not defined	Not measured.	Decreased liver enzyme levels. May improve HCV-associated clinical symptoms.	[111]
ICOMATRIX-adjuvanted recombinant core protein (Genotype 1a)	Healthy patients used.	Induced HCV-specific immune responses and CD+ T-cell responses in a subset of patients.	Clinical trial in healthy adults, no HCV infected patients tested.	[117] [118] [119]
MVA expressing NS3, NS4 and NS5B proteins (Transgene TG4040). (Genotype 1b)	Genotype not defined	Boosted HCV-specific immune responses	Transient decrease in viral load in a subset of patients.	[108] [120] [121]

* Cohort consisted of healthy volunteers, and recovered patients in addition to HCV infected patients. Genotype refers to viral genotype in the HCV infected patients only.
